# Surgical excision of osteochondroma on mandibular condyle via preauricular approach with zygomatic arch osteotomy

**DOI:** 10.1186/s40902-017-0129-x

**Published:** 2017-10-25

**Authors:** Sang-Hoon Park, Jun-Hyeong An, Jeong Jun Han, Seunggon Jung, Hong-Ju Park, Hee-Kyun Oh, Min-Suk Kook

**Affiliations:** Department of Oral and Maxillofacial Surgery, School of Dentistry, Dental Science Research Institute, Chonnam National University, 77, Yongbongro, Buk-Gu, Gwangju, 500-757 Korea

**Keywords:** Osteochondroma, 3D computer modeling, Minimally invasive

## Abstract

**Background:**

Osteochondroma is a benign tumor that tends to develop in mandibular condyle and coronoid process in the craniofacial region. If tumor mass has grown from condyle into the infratemporal space with zygomatic arch obstructing the access, there are risks associated with surgical exposure and local resection of these masses.

**Case presentation:**

This study reports on a case of osteochondroma on mandibular condylar head where we treated with surgical excision via preauricular approach with 3D analysis. After the local resection, there were no surgical and post-operative complications until 8-month follow-up period.

**Conclusions:**

In local excision of osteochondroma, our method is a minimally invasive method. It is a good example of osteochondroma treatment.

## Background

**Fig. 1:**
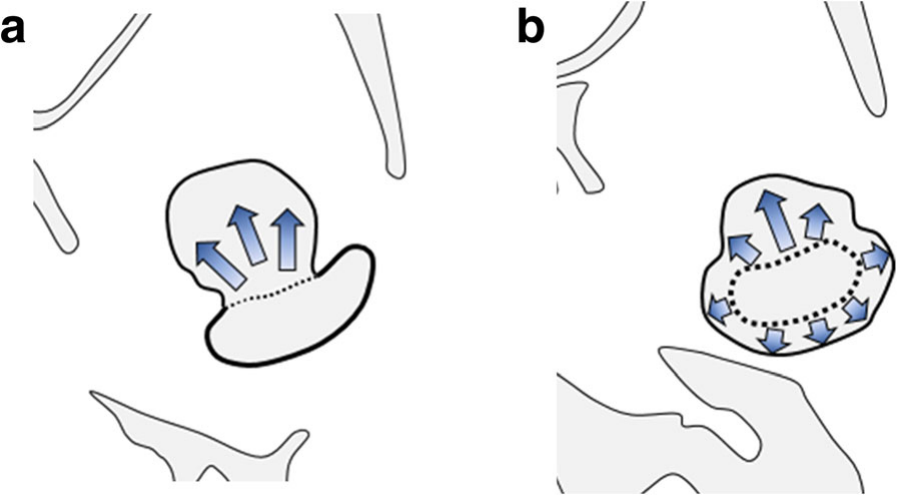
Classifications of osteochondroma. **a** Type 1 osteochondroma–protruding expansion. **b** Type 2 osteochondroma–globular expansion

Osteochondroma is characterized by cartilage-capped osseous protrusion from the external surface of the affected bone [1]. It can potentially occur at any site of endochondral ossification. In the craniofacial region, mandibular condyle and coronoid process are the most common affected sites. Some authors have classified this tumor in mandibular condyle into two types: type 1, a protruding expansion with mean proliferation direction of mass in one way, and type 2, a globular expansion with mean mass proliferate in all directions (Fig. 1) [2]. Almost all osteochondroma in mandibular condyle is type 1 [2]. The present case was also type 1.

If tumor mass has grown into the infratemporal space with zygomatic arch obstructing the access, there are risks associated with surgical exposure and local resection of these masses [3]. To reduce such risks, we visualized tumor mass and surrounding structures using 3D computer modeling program. With 3D CT work-up, obstructing structures could be identified with clear tumor margins so that we could plan small osteotomy of zygomatic arch with precise resection of the tumor mass.

Condylar osteochondroma can be treated with total condylectomy, subtotal condylectomy, conservative condylectomy, or local excision [4–7]. Traditionally, total condylectomy and subtotal condylectomy need submandibular and preauricular incision. Conservative condylectomy also needs preauricular incision with extension to the temporal area. However, with 3D CT findings, tumor size, and position can be confirmed precisely. Therefore, local excision with only small preauricular incision was needed in this case.

This study reports a case of osteochondroma on mandibular condylar head which was treated with local excision via preauricular approach and zygomatic arch osteotomy.

## Case presentation

**Fig. 2:**
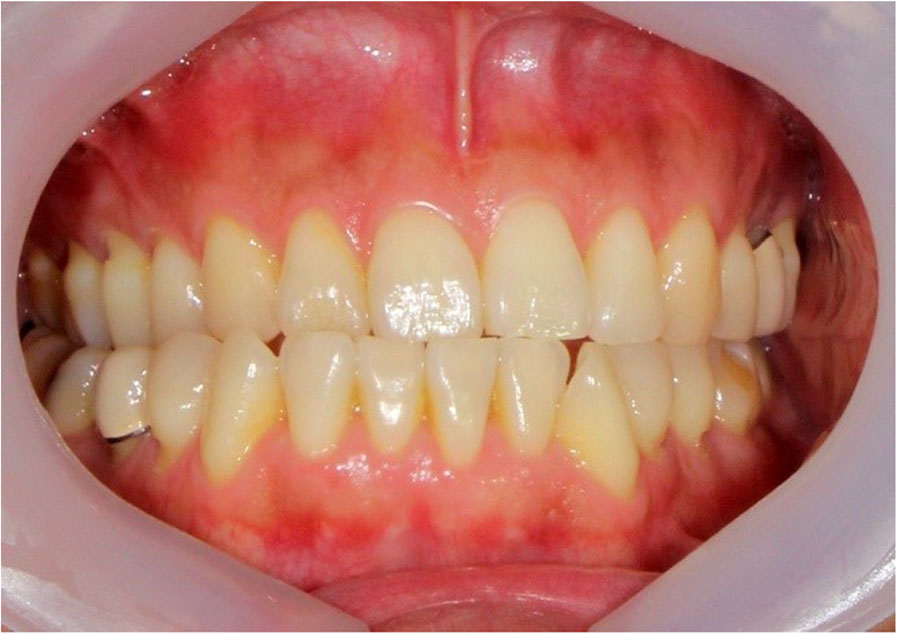
Menton is deviated to the right side. Dental *midline* is shifted to the *right side*

**Fig. 3:**
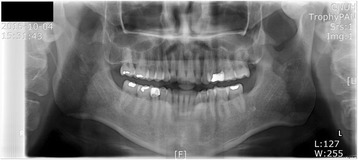
Proliferation of the *left* condylar head could be easily checked

A 46-year-old man was referred to our clinic due to chief complaints of chin deviation to the right side. There were right posterior cross bite and dental midline deviation to the right side by 5 mm (Fig. 2). In panorama radiograph, bony exophytic proliferation of the condyle was observed (Fig. 3). With additional imaging of CT and three-phase bone scan, tentative diagnosis of osteochondroma or osteoma on the left mandibular condyle was made.

### 3D CT work-up

**Fig. 4:**
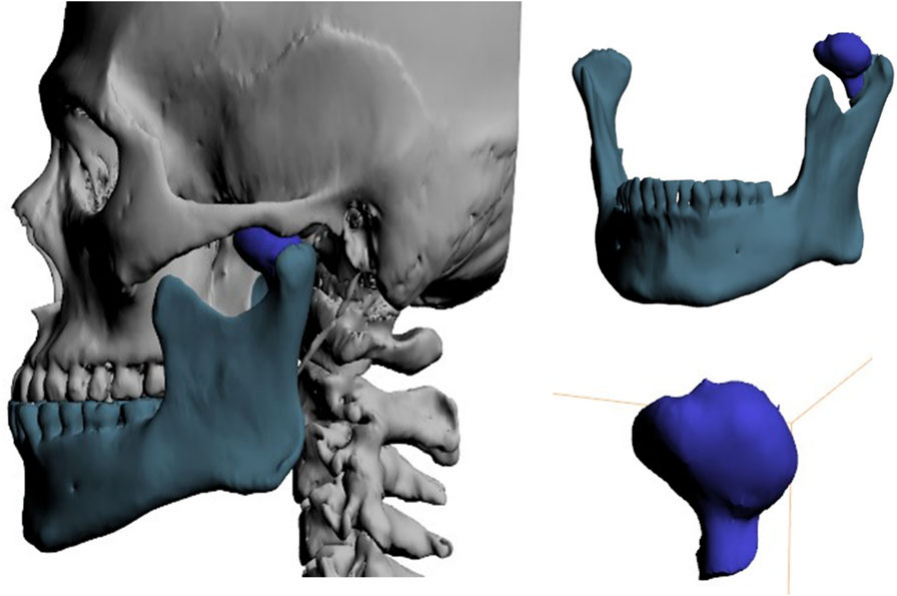
Reconstruction of three-dimensional image was done

Reconstruction and differentiation of tumor mass and surrounding structures were done using Mimics (Materialize NV, Leuven, Belgium). In a mandible only view, tumor mass could be observed at all directions. Resection line of tumor mass was identified (Fig. 4). We could anticipate that zygomatic arch obstructed access to the resection line and removal of the resected mass; thus, we plan zygomatic arch osteotomy. The tumor mass was about 20 × 20 × 15 mm; the size of zygomatic arch osteotomy was enough to be 15 mm for drawing out the resected mass.

### Surgical procedure

**Fig. 5:**
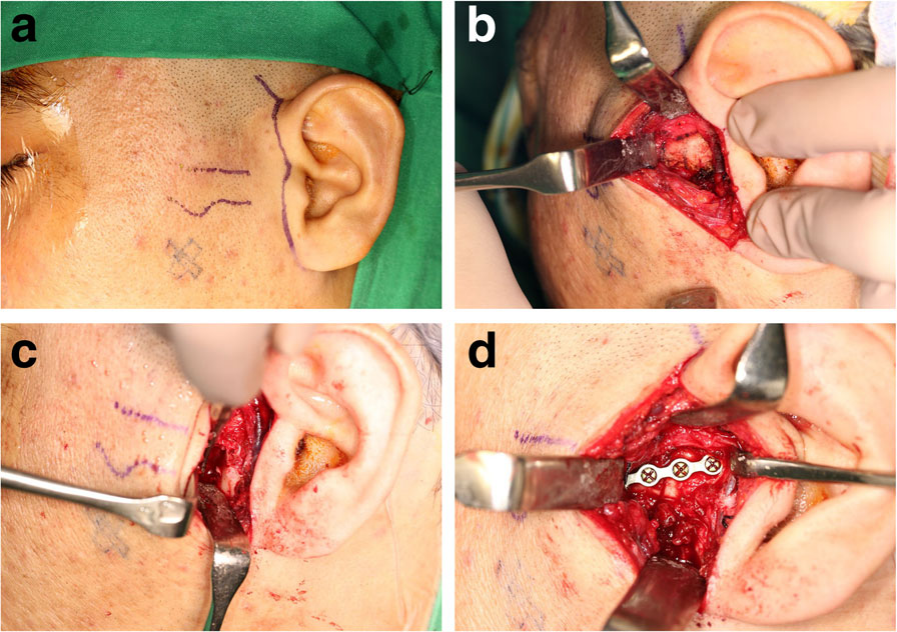
**a** Drawing of incision line. **b** Exposure of zygomatic arch. **c** Osteotomy of zygomatic arch. **d** Reposition of the zygomatic arch fragment after resection of tumor mass

**Fig. 6:**
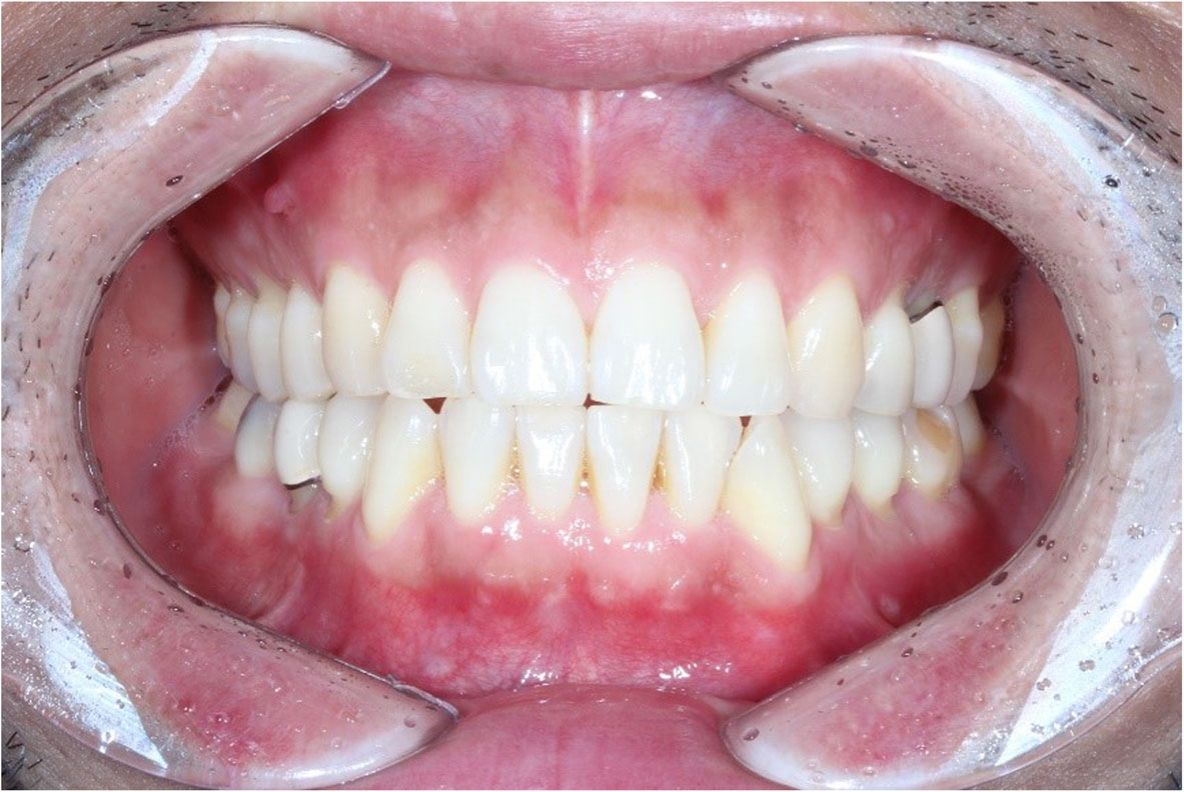
Dental *midline* is corrected after the surgery

Under general anesthesia, a preauricular incision was made. Dissection and bleeding control was done until zygomatic arch and temporomandibular joint capsule appeared. Two osteotomy lines were made. A posterior one was made at the foremost point of articular eminence while an anterior one was made at 15 mm in front of the posterior line. Zygomatic arch osteotomies were completed, and the fragment was displaced downward with the masseter muscle still attached. The joint capsule was incised, and the lateral pterygoid muscle was dissected to expose the tumor mass. Tumor mass was resected with saw. The surface of condyle was smoothed with round bur. Tumor mass was sent to the Department of Pathology. The zygomatic arch fragment was repositioned and fixed with a four-hole straight shape miniplate (Fig. 5). Finally, normal occlusion and proper midline relationship were checked (Fig. 6).

After the operation, intermaxillary fixation was done for 2 weeks with rubber rings. A month later, normal occlusion with proper dental midline was maintained (Fig. 6).

**Fig. 7:**
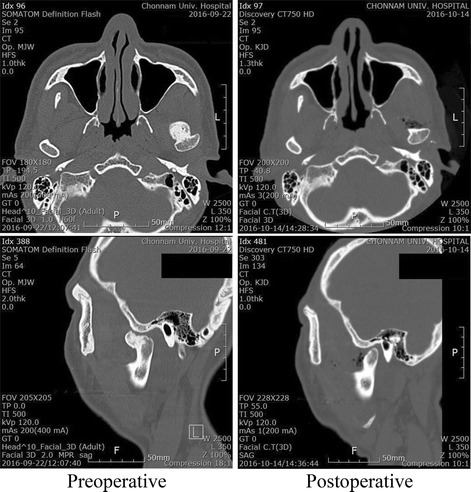
Preoperative CT scan (*left*) and postoperative CT scan (*right*)

CT was taken immediately after the operation. It confirmed that the tumor mass was removed (Fig. 7).

**Fig. 8:**
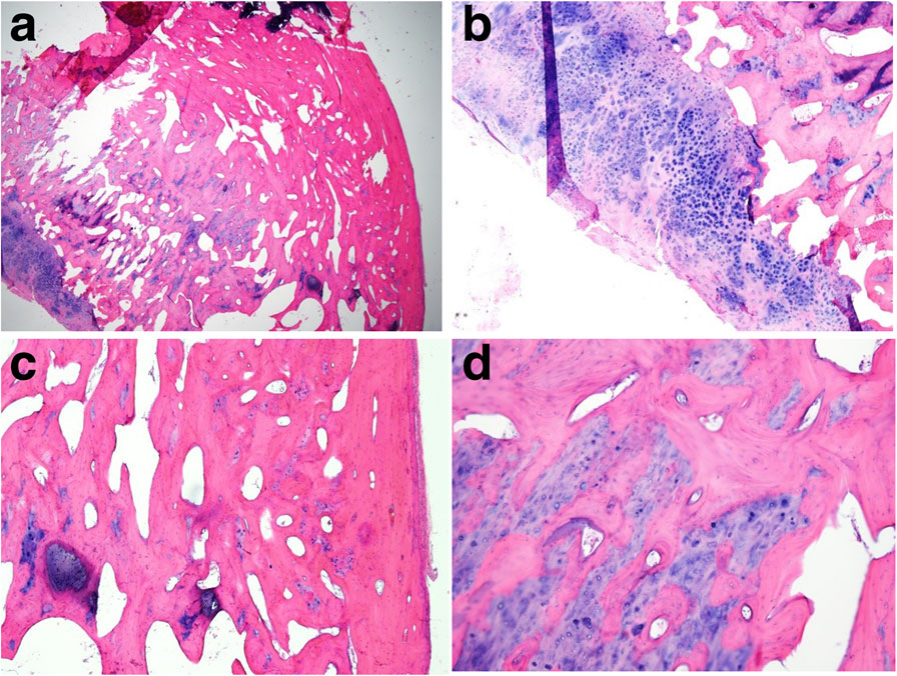
Histopathologic findings of the tumor mass. **a** × 10, cartilaginous cap could be identified. **b** × 20, head of the condyle. **c** × 40, endochondral ossification progressing beneath the cartilaginous cap. **d** × 100, hyaline cartilage gradually changed into trabecular bone

Pathologic diagnosis of the tumor mass was the osteochondroma (Fig. 8).

### Discussion

There are many theories about the etiology and pathogenesis of cartilage-capped and exophytic bony growths. They can occur in bones formed by endochondral ossification. They can also develop from displacement of the lateral portion of the growth plate which then proliferates in a direction diagonal to the long axis of the bone and away from the nearby joint [8]. In our patient, exophytic projection was extruded from the lateral portion of the condyle head with a direction diagonal to the long axis of the condyle. The peripheral part of the physis has been considered as a hernia from the growth plate [9]. This hernia may be idiopathic or traumatic. Regardless of the cause, the result is an abnormal extension of metaplastic cartilage responding to factors that cause exostosis growth by stimulating the growth plate. Development of these tumors in the mandibular condyle tends to support the theory that epiphyseal cartilage is idly located on the bone surface. It has been suggested that stress in the tough insertion region where local accumulation of cartilage dislocation is present can induce the formation of these tumors [2]. This could explain the fact that in the mandible, these lesions often arise at the coronoid process attached to temporalis and anteromedial condylar region attached to lateral pterygoid muscle insertion. Some authors also believe that trauma might play a role in the formation of these lesions [1].

Histopathological findings are crucial to the diagnosis of osteochondroma. Histologic examinations include a cartilaginous cap similar to that seen in a normal cartilage, endochondral ossification, cartilaginous islands in the subcortical bone, and a marrow space contiguous with the underlying bone (Fig. 8). It has been reported that the cartilaginous cap might be 10 mm or greater in thickness in the axial skeleton [10]. However, it tends to be thinner in the maxillofacial region. It might be absent in long-standing cases [10].

When considering treatment, the growing state and the type of osteochondroma are crucial. Growth status can be judged by repeated occlusal evaluation or bone scintigraphy. If an active growth is noticed in a child and the asymmetry is large, subtotal condylectomy is usually performed. However, in adult patient with the same symptoms, both side condylectomy and orthognathic surgery should be included. After such condylectomy, lateral open bite on the contralateral side might occur unless some kind of reconstruction is performed. A costochondral graft or a total stock joint prosthesis may be used. However, it has disadvantages such as exploration of the second surgical site, donor site morbidity, and bone resorption. A total joint prosthesis has disadvantages of high cost, material wear and potential failure, and restricted use in the growing patient [11, 12]. An alternative method might be vertical osteotomy of the ramus and advancing it superiorly to form a new condyle underneath the disc as described previously [13]. Locally derived bone graft attached to the medial pterygoid muscle has been utilized [14]. Some authors have proposed conservative condylectomy with less complications [15]. This protocol is applicable to osteochondromas involving the head of the condyle without extension of tumor into the neck. In patients with osteochondroma, the condylar head usually enlarges fairly and the neck of the condyle is significantly thickened [13]. Such neck thickening makes it possible to reproduce the remaining condylar stump for functioning as a “new” condylar head. The articular disc is then repositioned onto the “new” condyle and stabilized [13].

If the condition is inactive and there is no TMJ symptom, the reason for surgical intervention can be cosmetic or associated with dysfunction of mastication. In this situation, treatment can be chosen depending on osteochondroma classification. In type 2 with globular expansion, condylectomy should be performed as described above. In type 1 with protruding expansion, just local excision is sufficient. Some excellent outcomes have been reported for type 1 [12]. In this study, the patient’s age was 37 years old. There was a mild bone activity on the condyle head in the three-phase bone scan. Exophytic bony protrusion area was approximately 2 × 2-cm sized, heading for anteromedial of the condyle. It was type 1 osteochondroma. Based on these facts, we decided to do local excision.

After 3D reconstruction of the CT images, the tumor mass was growing to anteromedial side of the mandibular condyle; thus, it might be difficult to approach to the mass through only preauricular incision due to the obstruction of zygomatic arch (Fig. 4). To allow the access of cutting instruments to the anteromedial side of the mandibular condyle and removal of the excised tumor without extension of incision or dissection, we planned zygomatic arch osteotomy over the tumor mass. During the surgery, zygomatic arch osteotomy provided easy access to anteromedial portion of the condyle without additional incision or wide dissection, and tumor mass was drawn out with only 4 cm of preauricular incision with zygomatic arch osteotomy. Though minimally invasive procedure in this study cannot be applied in all osteochondroma cases, it can be one of the simple treatments for not-growing type 1 osteochondromas.

The malignant potential and risk of recurrence are the major drawbacks of conservative procedure. The recurrence rate for solitary osteochondromas in long bone is approximately 2% [16]. Of all the condylar osteochondromas reported, three have shown recurrence [15, 17, 18]. For the three cases, excision and condyloplasty rather than condylectomy were done as the initial procedure [1]. Because of a short follow-up period, we cannot evaluate recurrence for this case. However, based on mild bone activity in three-phase bone scan and other cases in the literature, it is expected that there will be no recurrence.

## Conclusions

Osteochondroma in the facial area is an uncommon disease. However, more cases have been reported and its treatment has been systematized. Its growth state and the type of tumor are important to decide treatments. In the case of inactive type 1 osteochondroma, local excision alone can provide good results. In local excision of osteochondroma, our method is a minimally invasive method. It is a good example of osteochondroma treatment.
